# Comparative genomics study of *Staphylococcus aureus* isolated from cattle and humans reveals virulence patterns exclusively associated with bovine clinical mastitis strains

**DOI:** 10.3389/fmicb.2022.1033675

**Published:** 2022-11-07

**Authors:** Romário Alves Rodrigues, Lucas José Luduverio Pizauro, Alessandro de Mello Varani, Camila Chioda de Almeida, Saura Rodrigues Silva, Marita Vedovelli Cardozo, Janet I. MacInnes, Andrew M. Kropinski, Poliana de Castro Melo, Fernando Antonio Ávila

**Affiliations:** ^1^Department of Reproduction Pathology and One Health, Faculty of Agricultural and Veterinary Sciences, São Paulo State University, Jaboticabal, São Paulo, Brazil; ^2^Department of Agricultural and Environmental Sciences, Santa Cruz State University, Ilhéus, Bahia, Brazil; ^3^Department of Agricultural and Environmental Biotechnology, Faculty of Agricultural and Veterinary Sciences, São Paulo State University, Jaboticabal, São Paulo, Brazil; ^4^Laboratory of Microorganism Physiology, Minas Gerais State University, Passos, Minas Gerais, Brazil; ^5^Department of Pathobiology, Ontario Veterinary College, University of Guelph, Guelph, ON, Canada; ^6^Department of Food Science, University of Guelph, Guelph, ON, Canada

**Keywords:** biofilm formation, public health, virulence genes, adhesins, phylogeny, antimicrobial resistance

## Abstract

*Staphylococcus aureus* causes nosocomial and intramammary infections in humans and cattle, respectively. A large number of virulence factors are thought to play important roles in the pathogenesis of this bacterium. Currently, genome-wide and data-analysis studies are being used to better understand its epidemiology. In this study, we conducted a genome wide comparison and phylogenomic analyses of *S. aureus* to find specific virulence patterns associated with clinical and subclinical mastitis strains in cattle and compare them with those of human origin. The presence/absence of key virulence factors such as adhesin, biofilm, antimicrobial resistance, and toxin genes, as well as the phylogeny and sequence type of the isolates were evaluated. A total of 248 genomes (27 clinical mastitis, 43 subclinical mastitis, 21 milk, 53 skin-related abscesses, 49 skin infections, and 55 pus from cellulitis) isolated from 32 countries were evaluated. We found that the *cflA, fnbA, ebpS, spa, sdrC, coa, emp, vWF, atl, sasH, sasA*, and *sasF* adhesion genes, as well as the *aur, hglA, hglB,* and *hglC* toxin genes were highly associated in clinical mastitis strains. The strains had diverse genetic origins (72 protein A and 48 sequence types with ST97, ST8 and ST152 being frequent in isolates from clinical mastitis, abscess, and skin infection, respectively). Further, our phylogenomic analyses suggested that zoonotic and/or *zooanthroponotic* transmission may have occurred. These findings contribute to a better understanding of *S. aureus* epidemiology and the relationships between adhesion mechanisms, biofilm formation, antimicrobial resistance, and toxins and could aid in the development of improved vaccines and strain genotyping methods.

## Introduction

*Staphylococcus aureus* is a commensal microorganism that can cause several important diseases in humans and animals. It is considered a facultative intracellular pathogen that is responsible for recurrent infections (Watkins and Unnikrishnan, 2020). In humans, its primary entry points are damaged skin and mucosa, which can lead to skin infections, septicemia, endocarditis, and abscesses (Lalaouna et al., 2018). In cattle, intramammary *S. aureus* infections (clinical and subclinical mastitis) spread primarily *via* milking machines, milkers’ hands, and vectors such as flies (Zadoks et al., 2011).

Virulence factors are important in the development, duration, and severity of *S. aureus* related illnesses since they are often involved in host defense evasion (Wójcik-Bojek et al., 2022). Adhesins play a role in cell attachment, invasion, and biofilm formation (Kerro Dego, 2020), thus allowing for increased persistence and protection against antimicrobial factors in the host (Gajewska and Chajęcka-Wierzchowska, 2020). The production of toxins such as hemolysins, leukotoxins, proteases, and other enzymes can cause direct host cell injury (Otto, 2014). *S. aureus* can also carry a large number of resistance genes which pose a serious economic threat to the dairy sector and to public health (Igbinosa et al., 2016).

The contamination of food products by *S. aureus* can be aided by virulence factors that mediate the interaction of pathogen with the host and the environment (Gajewska and Chajęcka-Wierzchowska, 2020; Bencardino et al., 2021). When a dairy herd has clinical mastitis (animals with obvious disease) or subclinical mastitis (animals with no obvious signs) milk contamination and discard rates increase, productivity decreases, antimicrobial treatment costs increase, and animals may have to be culled prematurely (Exel et al., 2022). Zoonotic transmission of *S. aureus* has been widely documented (Patel et al., 2021) and characterization of sequence types (STs) helps to elucidate strain relationships and epidemiology, as well as gene transfer amongst strains from different hosts (Bruce et al., 2022).

Despite the importance of *S. aureus* in human and veterinary medicine there is a dearth of studies on gene profile, strain-to-strain relationships, disease-host relationships, and host-pathogen interactions (Pizauro et al., 2021). In the current study we used genome wide analyses for genome comparison to better understand strain-to-strain relationships, disease-host relationships, and host-pathogen interactions. Comparative genomics and phylogenomic analyses were used to characterize the adhesion, biofilm, toxin, and antimicrobial resistance genes of *S. aureus* isolated from human and cattle samples.

## Materials and methods

### Bacterial sequencing and origin of isolates

The National Center for Biotechnology Information database (NCBI; Clark et al., 2016) was searched for *S. aureus* genomes of bovine (*Bos taurus*) and human (*Homo sapiens*) origin. For this search, we used “organism” from the “Available Facets” section of the NCBI’s web platform Sequence Set Browser, and then the microorganism “*Staphylococcus aureus*” from the “Top Organisms” section. The *S. aureus* genomes from *Bos taurus* were analyzed, in addition to raw milk/bulk milk, and the condition (clinical mastitis, subclinical mastitis) was described as a stage of the disease in the BioSample description or in scholarly publications. *S. aureus* genomes of isolates from *Homo sapiens* that had abscess, skin infection, or pus listed as the source of isolation in the BioSample description or academic publications were then obtained; in addition, other sources of isolation such as surgical site, skin, and wound secretion were accepted for the group “skin infections.” Genome sequences deposited from June 2020 until January 2021 were analyzed.

### Genomic annotation: Identification of adhesin, biofilm formation, toxin, and antimicrobial resistance genes

*S. aureus* genomes were annotated using Rapid Annotation using Subsystem Technology (RAST; Aziz et al., 2008). The identification of genes related to adhesion and biofilm formation were classified based on the results of the RAST platform and the Virulence Factor of Pathogenic Bacteria (VFDB) reference database (Chen et al., 2005). The antimicrobial resistance genes were annotated using ResFinder 4.1 and toxin genes with VirulenceFinder 2.0, both from the Center of Genomic Epidemiology (Bortolaia et al., 2020).

### Pan-genome determination

To normalize the analyses, all genomes were annotated using Prokka 1.14.6 (Seemann, 2014). The computational tool Roary 3.13.0 was used to infer the pangenome (Page et al., 2015). The Roary tool was also used to calculate pangenome size and reveal orthologous genes.

### Phylogenomics analysis

The phylogenomic concatenated matrix was created with PRANK (Löytynoja, 2014) using codon-aware alignment composed of 247 genes shared across all analyzed *Staphylococcus* spp. genomes. The phylogenetic tree was constructed using the maximum likelihood (ML). The ML tree was calculated using IQ-Tree 2 (Minh et al., 2020) with the best-of-fit model GTR + F + R6 according to AIC criteria (Akaike, 1974), with the software tool ModelFinder (Kalyaanamoorthy et al., 2017). Clade support was estimated using the ultrafast bootstrap (UFBoot) and SH-aLRT algorithms (Hoang et al., 2018) with 1.000 replicates. Tree rooting was based on the *Escherichia coli* genomes (GenBank access numbers: CP058682, NZ CP037943, NZ CP027390, NZ AP018808, and NZ AP018808) and tree drawing was done using Interactive tree of life (iTOL) v6 (Letunic and Bork, 2016).

### *In silico* determination of multilocus sequence typing and *spa*

Multilocus sequence types were determined *in silico* using the MLST 2.0 online platform (Larsen et al., 2012), and *spa* typing was done using spaTyper version 1.0 (Bartels et al., 2014), both from the Center of Genomic Epidemiology.

### Statistical analyzes

Statistical analyzes were performed using the R program 4.1.2 (R Core Team, 2017). The Fisher’s exact test was used to analyze possible associations between the presence of each of the virulence genes (gene by gene) in each type of disease evaluated, using a contingency table considering “1” as presence and “0” as absence and a significant value of *p* < 0.05. Multinomial logistic regression was done with all genes that showed a significant value in Fisher’s test to assess the significance of association between the types of condition, considering raw milk samples as the reference, and the origin of the isolates being the response variable, and the genes, the explanatory variable. Using a modification of the method of Åvall-Jääskeläinen et al. (2021) the multinomial logistic regression model was then analyzed by Wald’s Z test and the value of *p* values were obtained by the pnorm() function of the nnet package. Genes with p < 0.05 were considered significant for the determination of the model in each disease (Dalgaard, 2008).

Multinational logistic regression model used was:


$$logit\left(\frac{Source_{\left[rj\right]}}{Source_{rj6}}\right)=\beta_{0c}+\beta_{1c}\mathrm{Screenedvirulencegenes}{\left(\mathrm{adhesion}/\mathrm{toxins}/\mathrm{resistance}\right)}_{r}+\beta_{2c}{\mathrm{MLST}}_{j}$$

*β*_0c_ represents the medium value for the category _c_ of the response variable*β*_1c_ Screened virulence genes, represent the fixed effects of the Virulence genes category with c as *r* = 1,---,36;*β*_2c_ MLST, represent the fixed effects of the variable MLST category with c as *j* = 1---,26.*c* = 1,--5, represent the 5 *logit* functions that will be used when the Raw milk is used as a baseline for comparison of each source.

Evaluation between the association of the presence of adhesion and biofilm genes and the origin of isolation was done using the Spearman correlation test (Dalgaard, 2008). The correlation coefficients and the gene frequency by origin were presented in heat maps.

### Genome-wide association test accounting population structure

The treeWAS R package https://github.com/caitiecollins/treeWAS (Collins and Didelot, 2018) was used to apply a phylogenetic tree-based approach to the genes and genome studied. For these analyses, a genetic dataset with a matrix containing binary genetic data (gene presence/absence), a phenotypic variable (isolates isolation source as a continuous numeric value) and the phylogenetic tree consisted of the 247 genes that were shared among all isolates was used. These findings were further assessed with the multinomial logistic regression.

## Results

### Genome and pangenome assessment

**Figure 1:**
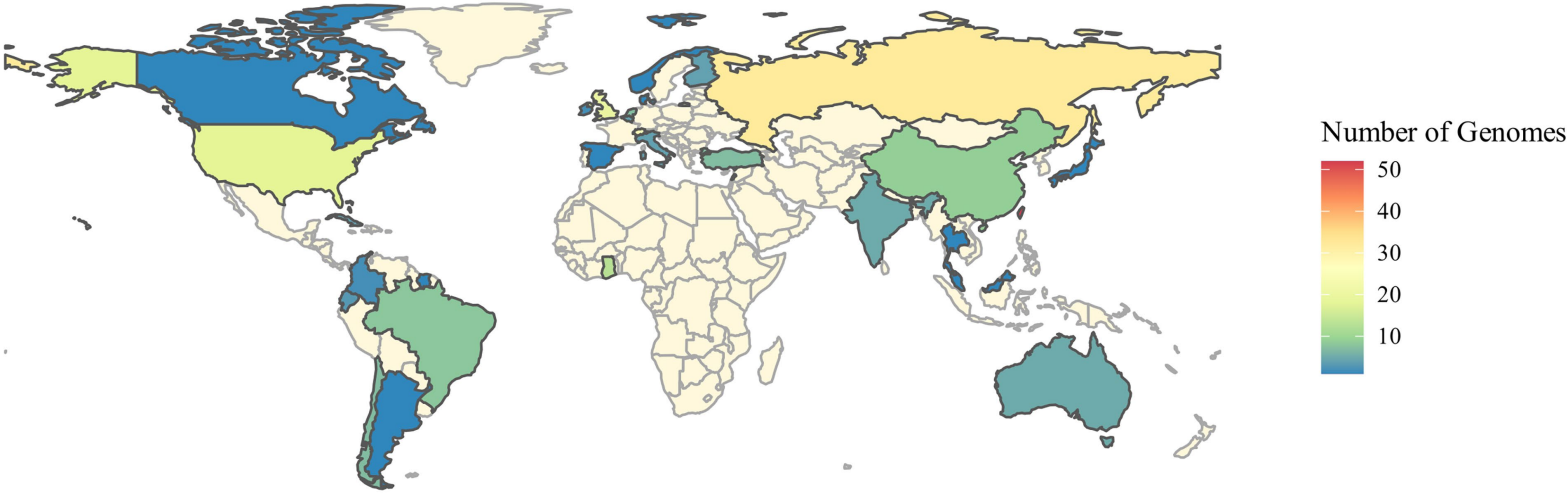
Spatial distribution of the isolates according to the information presented in the NCBI GenBank.

The genomes of 248 independent strains of *S. aureus* from six different sources: diseased cattle (27 clinical mastitis and 43 subclinical mastitis cases); diseased human (53 skin-related abscesses, 49 skin infections, and 55 pus from cellulitis cases) and healthy controls (21 raw milk/bulk milk) were evaluated. The isolates came from 32 countries, i.e., Argentina (1), Australia (5), Bahamas (1), Belgium (4), Brazil (8), Canada (1), Chile (7), China (9), Colombia (2), Cuba (4), Denmark (1), Ecuador (3), Finland (4), Ghana (13), India (5), Ireland (2), Italy (4), Japan (1), Lebanon (7), Malaysia (1), Netherlands (7), Norway (1), Russia (32), South Sudan (1), Spain (1), Suriname (1), Switzerland (24), Taiwan (52), Thailand (1), Turkey (7), United Kingdom (20) and United States (18) (Figure 1).

**Figure 2:**
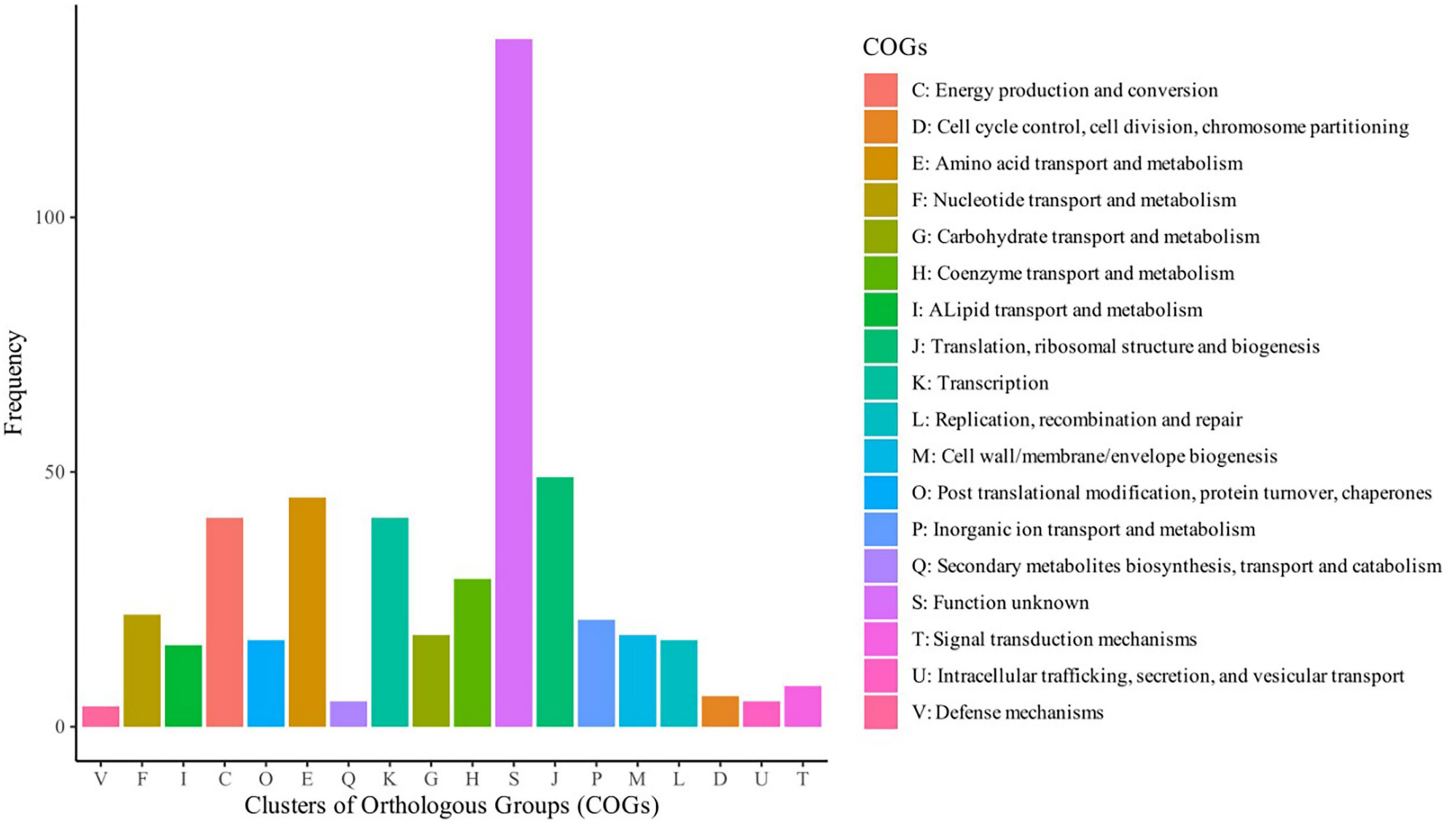
Category of Clusters of Orthologous Groups (COG) of genes central to the genomes of 248 *Staphylococcus aureus* isolated from humans (abscesses, skin infections and pus) and from cattle (clinical mastitis, subclinical mastitis and raw milk).

**Figure 3:**
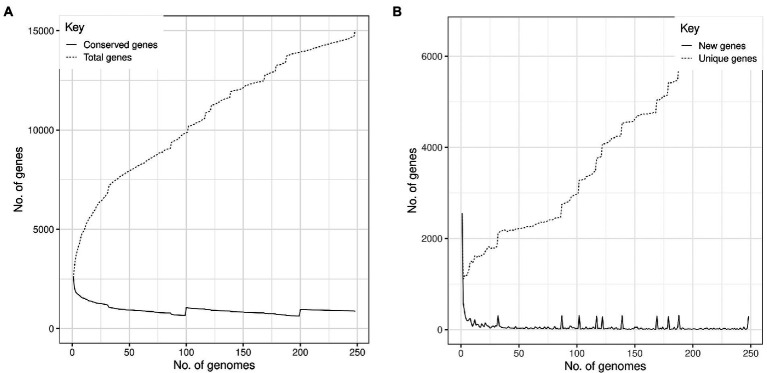
*Staphylococcus aureus* pangenome profile isolated from humans (abscesses, skin infections, and pus) and from cattle (clinical mastitis, subclinical mastitis, and raw milk). **(A)** The number of genes as a function of the number of genomes. The solid line indicates the number of conserved genes in the core genome and the dashed line the total number of genes. **(B)** The number of new pangenome genes as a function of the number of analyzed genomes. The solid line indicates the number of new genes and the dashed line, the total number of unique genes.

Based on assessment of these 248 *S. aureus* genomes, the pangenome analysis revealed 15,011 accessory genes and 873 genes belonging to the central genome. Five hundred and ninety-four genes were classified as soft-core genes (i.e., shared between 95 and 98% of the identified genomes); 1,842 as shell genes (shared between 15 and 95% of the genomes) and 11,702 as cloud genes (shared by less than 15% of the analyzed isolates). Central genome genes were associated with translation, ribosomal structure and biogenesis (9.86%), amino acid transport and metabolism (9.05%), transcription (8.25%), energy production and conversion (8.25%); many others were of unknown function (27.16%; Figure 2). The increase in the number of single genes was inversely related to the number of new genes revealed for this species (Figure 3).

### Distribution of adhesin, biofilm, antimicrobial resistance, and toxin genes in *S. aureus* isolates

Of the 248 *S. aureus* genomes studied, 70 were bovine disease isolates, 157 were from humans with skin associated disease, and 21 were from raw milk. Annotation using RAST identified 26 adhesin genes (*clfA, clfB, cna, fnbA, fnbB, ebpS, spa, sdrC, sdrD, sdrE, coa, eap, emp, efb, vWbp, atl, aap, pls, sasG, sasH, sasA, sasC, sasD, sasF, sasI, sasK*) and nine biofilm formation genes (*icaA, icaB, icaC, icaD, icaR*, *rbf*, *tcaR*, *sarA* and *sigB*). Thirty antimicrobial resistance genes (*adD, aac(6′)-aph(2″), aadE, ant(9)-Ia, aph(3′)-III, bla*TEM-116*, blaZ, cat (pC233), dfrG, erm*(A)*, erm*(B), *erm*(C)*, fosB4, fosB6, fusB, fusC, lnu* (A)*, lnu* (B), *Isa* (E), *mecA, mecA1, mph*(C), *msr*(A*), mup*(A)*, sal* (A)*, str, tet*(K), *tet*(M)*, vga*(A)_V_ and *qacD*) were identified using ResFinder and 43 toxin genes (i.e., *aur, splA, splB, splE,* ACME*, sak, scn, edinB, hlgA, hlgB, hlgC, lukD, lukE, lukF-PV, lukS-PV, sea, seb, sec, sec3, sed, seg, seh, sei, sej, sel, sek, sem, sen, seo, sep, seq, seu, ser* and *tst*) were identified using VirulenceFinder (Supplementary Material 1).

**Figure 4:**
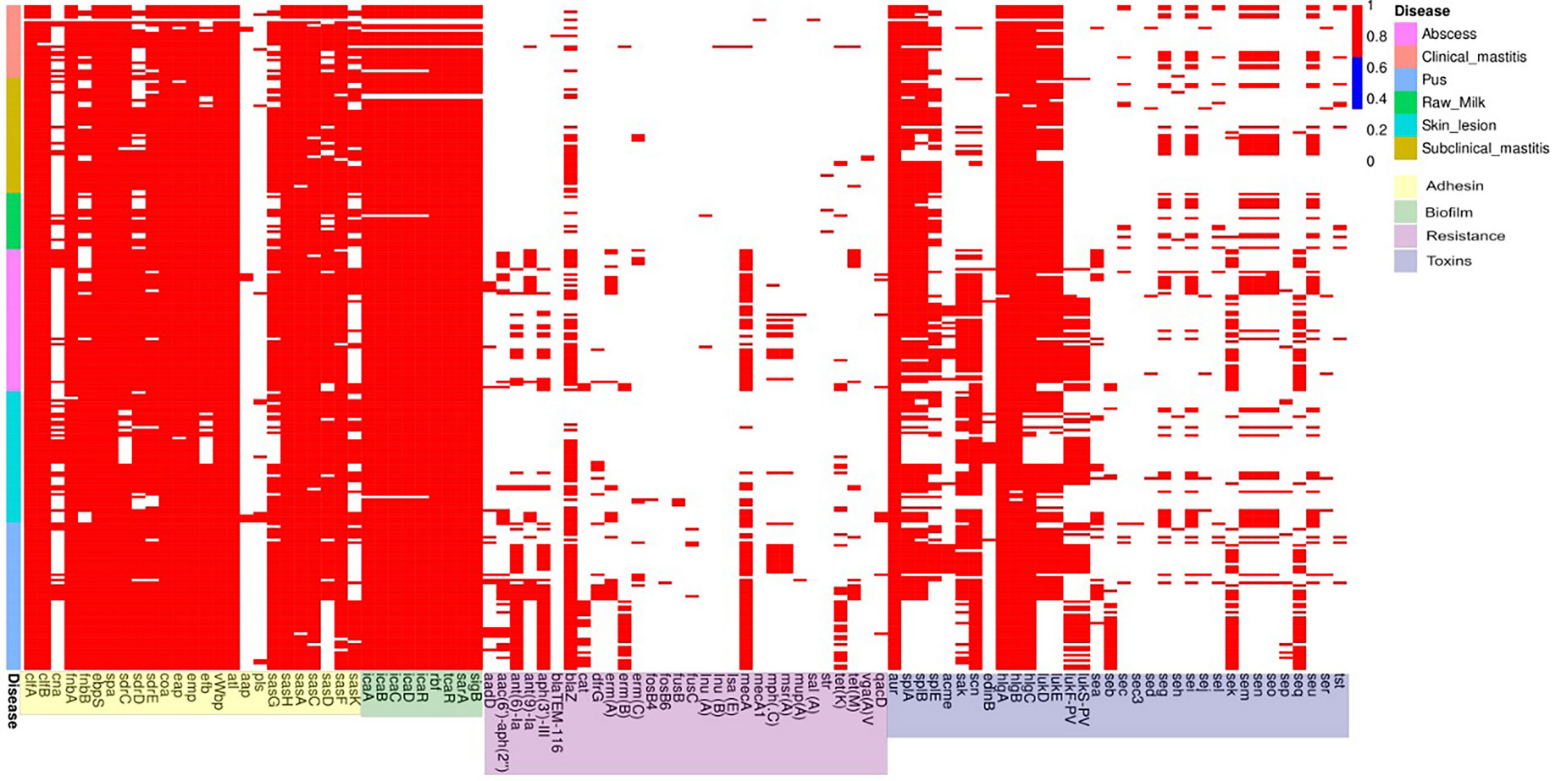
Binary frequency graph of adhesion, biofilm, antimicrobial resistance, and toxin gene frequencies in *S. aureus* from clinical and subclinical bovine mastitis cases and raw milk, and from human abscesses, skin infections, and pus.

Adhesin genes *clfA, clfB, fnbA, ebpS, spa, coa, eap, emp, vWbp, atl, sasH, sasA, sasC,* and *sasF* genes were observed at high frequencies (> 90%) in isolates from all sources while the *cna*, *aap* and *pls* genes were observed in fewer strains (~20%). Differences in the frequencies of some genes were seen (e.g., clinical mastitis genomes had a lower frequency, approximately 83%) of the *icaA*, *icaB*, *icaC* and *icaD* biofilm genes and biofilm regulator genes *rbf, tcaR, sarA* and *sigB* while in the other groups, these genes were present in >90% of the strains evaluated. There was a high prevalence (>90%) of the *fnbB* gene in skin and pus infection isolates and of the *sdrD* and *sasG* genes in abscesses and pus strains; both of which also presented similar frequencies in the bovine isolates. The *sdrE* and *efb* genes were less prevalent (< 90%) in isolates from clinical mastitis and skin infection cases; the *sdrC* gene in skin infection isolates, and *sasD* in clinical mastitis, raw milk, and pus isolates (Figure 4; Supplementary Material 2).

The antimicrobial resistance genes *aadD, aac(6′)/aph(2″), aadE, ant(9)-Ia, aph(3′)-III, cat(pC233), dfrG, erm(A), erm(B), mecA, mph(C), msr(A), tet(K)* and *tet(M)* were most prevalent in human isolates and only seen sporadically in bovine isolates. The *bla*TEM-116 *erm*(C), *fosB4, fosB6, fusB, fusC, lnu*(A), *lnu*(B), *lsa(E)*, *mecA1, mup*(A), *sal*(A), *str, vga*(A)_V_ and *qacD* genes were observed sporadically in all groups. The *blaZ* gene was present (albeit unevenly) in all groups while the *mecA* gene was detected only in bovine isolates; however, both were prevalent (> 90%) in pus isolates (Figure 4). The *aur*, *hlgA*, *hlgB* and *hlgC* toxin genes were detected in >90% of the isolates of the all groups; the prevalence of the *splA* gene in the clinical mastitis isolates and raw milk, *splB* in raw milk, and *scn* in pus isolates was also >90%. The *lukD* and *lukE* genes were also prevalent in isolates from raw milk and abscesses, and as well as *lukF-PV* and *lukS-PV*, they were equivalent to each other in almost all groups evaluated (Figure 4).

### Phylogenomics analysis

**Figure 5:**
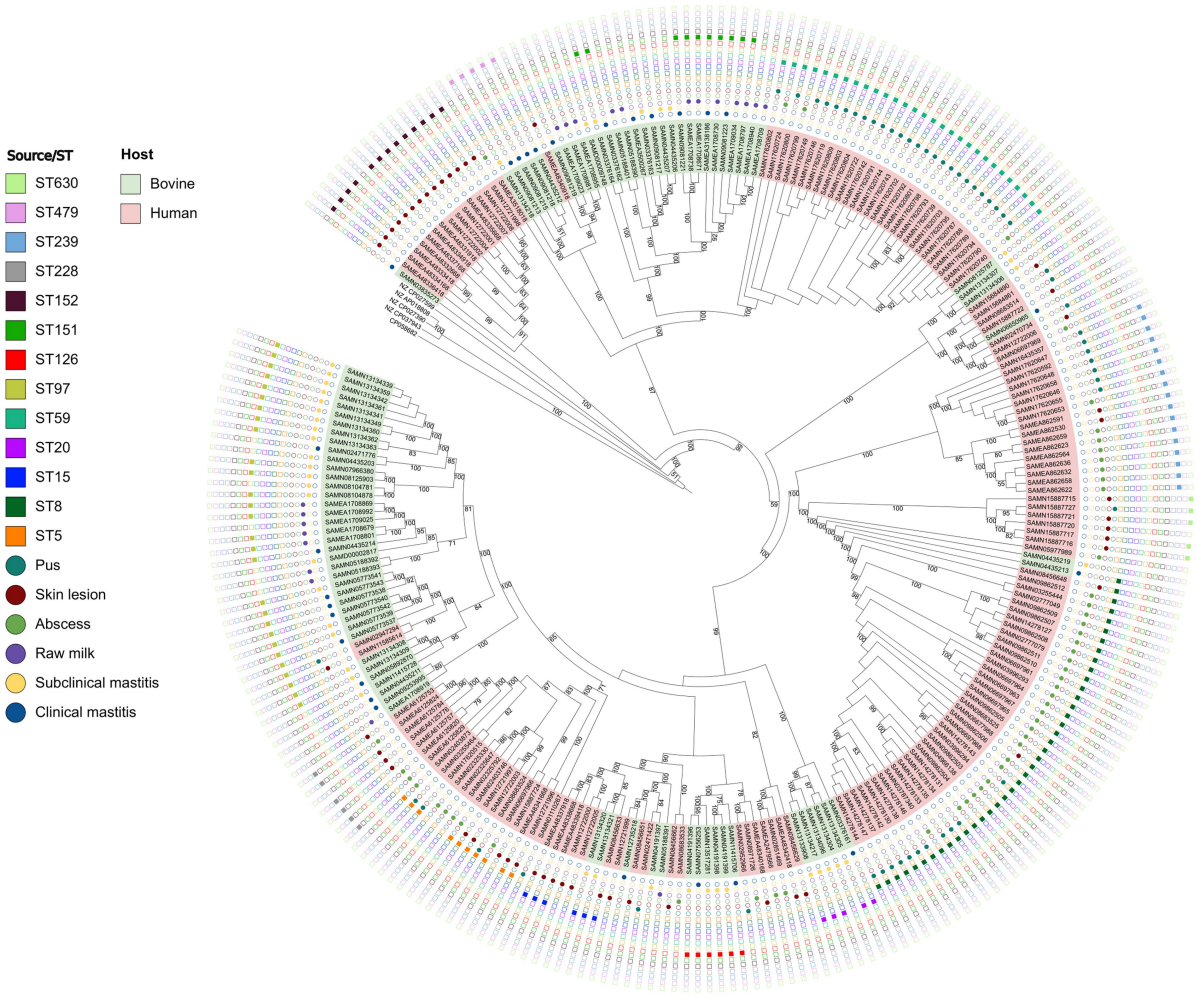
Phylogenomic tree based on 248 genes shared across all analyzed *Staphylococcus aureus* isolated from bovine clinical mastitis and subclinical mastitis cases and raw milk and from human abscesses, skin, and pus infections.

Phylogenetic analysis of the *S. aureus* isolates in this study revealed that the clinical and subclinical cattle isolates formed distinct clades consistent with zoonotic and/or *zooanthroponotic* transmission (Figure 5). Some STs were more relevant when considered their respective origins? In this regard, ST239 and ST5 isolates were only recovered from humans and ST151 only in bovine isolates. ST8 was more frequent in abscess isolates, with the ST97 in subclinical mastitis, ST59 in pus isolates and ST152 in skin infection. These findings reinforces the epidemiological importance of these STs (Figure 5; Supplementary Material 3).

### Multilocus sequence and *spa* typing

Forty-eight sequence types (ST) were assigned by MLST analysis. ST8 (15.7%), ST97 (12.1%), ST59 (11.7%), ST239 (6.85%), and ST152 (5.24%) were most prevalent. Eleven isolates could not be typed. The most common protein A (t) types as characterized by *spa* typing [i.e., t008 (*n* = 32), t437 (*n* = 26), t355 (*n* = 11), and t267 (*n* = 8)] were categorized according to source of isolation, host, and ST (Supplementary Material 3).

### Association between the presence of virulence genes and the origin of the isolate

Using the Fisher’s test as a screening step to test the significance of all genes based on their origin it was possible to observe that nine adhesins, nine biofilm, 18 resistance, and 25 toxin genes were significant (*p* < 0.05; Table 1).

**Table 1:** Significant gene profile by Fisher’s exact test and by multinomial logistic regression according to the origin of the isolate and the main sequence type.

Origin	Gene profiles	MLST
Clinical mastitis	**adhesins***: clfB,cna, fnbB, sdrC, sdrD, efb, sasG, sasD, sasK,* **biofilm**: *icaADBC, icaR, rbf, tcaR, sarA, sigB,* **resistance**: *aadD, aac(6′)-aph(2″), aadE, ant(9)-Ia, aph(3′)-III, cat (pC233), dfrG, erm(A), erm*(B), *fusB, fusC, mecA, mph*(C)*, msr*(A), *str, tet*(K), *tet*(M),**toxin***: splA, splB, splE,* ACME*, sak, scn, edinB, hlgC, lukD, lukE, lukF-PV, lukS-PV, sea, seb, sec, seg, sei, sel, sek, sem, sen, seo, seq, seu, tst*	ST97
Subclinical mastitis	**adhesins***: clfB,cna, fnbB, sdrC, sdrD, efb, sasG, sasD, sasK,* **biofilm**: *icaADBC, icaR, rbf, tcaR,sarA, sigB,* **resistance**: *aadD, aac(6′)-aph(2″), aadE, ant(9)-Ia, aph(3′)-III, cat (pC233), dfrG, erm*(A)*, erm*(B), *fusB, fusC, mecA, mph*(C)*, msr*(A)*, tet*(K), *tet*(M*),* **toxin***: splA, splE, acme, sak, scn, edinB, hlgC, lukD, lukE, lukF-PV, lukS-PV, sea, seb, sec, seg, sei, sel, sem, sen, seo, seq, seu, tst*	–
Abscess	**adhesins***: clfB, cna, fnbB, sdrC, sdrD, efb, sasG, sasD, sasK,* **biofilm**: *icaADBC, icaR, rbf, tcaR,sarA, sigB,* **resistance**: aadD, aac(6′)-aph(2″), *aadE*, ant(9)-Ia, aph(3′)-III, blaZ, *cat (pC233),* dfrG, erm(A), erm(B), mecA, mph(C), msr(A), str, tet(K),**toxin**: splA, splB, splE, acme, sak, scn, edinB, hlgC, lukD, lukE, lukF-PV, lukS-PV, sea, seb, sec, seg, sei, sel, sek, sem, sen, seo, seq, seu	ST8
Skin infection	**adhesins***: clfB, cna, fnbB, sdrC, sdrD, efb, sasG, sasD, sasK,* **biofilm**: *icaADBC, icaR, rbf, tcaR, sarA e sigB,* **resistance**: *aadD, aac(6′)-aph(2″), ant(9)-Ia, aph(3′)-III, blaZ, cat (pC233), dfrG, erm*(A)*, erm*(B), *fusB, fusC, mecA, mph*(C), *msr*(A)*, str, tet*(K),**toxin***: splA, splB, splE, acme, sak, scn, edinB, hlgC, lukD, lukE, lukF-PV, lukS-PV, sea, seb, sec, seg, sei, sel, sek, sem, sen, seo, seq, seu, tst*	ST152
Pus	**adhesins***: clfB, cna, fnbB, sdrC, sdrD, efb, sasG, sasD, sasK,* **biofilm**: *icaADBC, icaR, rbf, tcaR,sarA, sigB,* **resistance**: *aadD, aadE, ant(9)-Ia, aph(3′)-III, blaZ, cat (pC233), erm*(A)*, erm*(B), *fusB, fusC, mecA, mph*(C)*, msr*(A)*, str, tet*(K),**toxin***: splA, splB, splE, acme, sak, scn, edinB, hlgC, lukD, lukE, lukF-PV, lukS-PV, sea, seb, sec, seg, sei, sel, sek, sem, sen, seo, seq, seu, tst*	–

Multinomial logistic regression analysis showed that most genes had a significant relationship with the origin of the isolate, except for some antimicrobial resistance and toxin genes. The *blaZ* gene was the only antimicrobial resistance gene did not have a significant association with clinical mastitis isolates and, in conjunction the *str*, *splB* and *sek* genes, with subclinical mastitis isolates. The *fusB, fusC, tet(M)* and *tst* genes were not significantly associated with abscess isolates, nor were the *aadE* and *tet(M)* genes associated with skin infection isolates, or the *aac(6′)-aph(2′), drfG* and *tet(M)* genes to pus isolates. Multinomial logistic regression analysis also showed that there was significant relationships between origin and MLST with clinical mastitis (ST97), abscess (ST8) and skin infection isolates (ST152)., (Table 1; Supplementary Material 3).

### Correlation amongst adhesion genes

**Figure 6:**
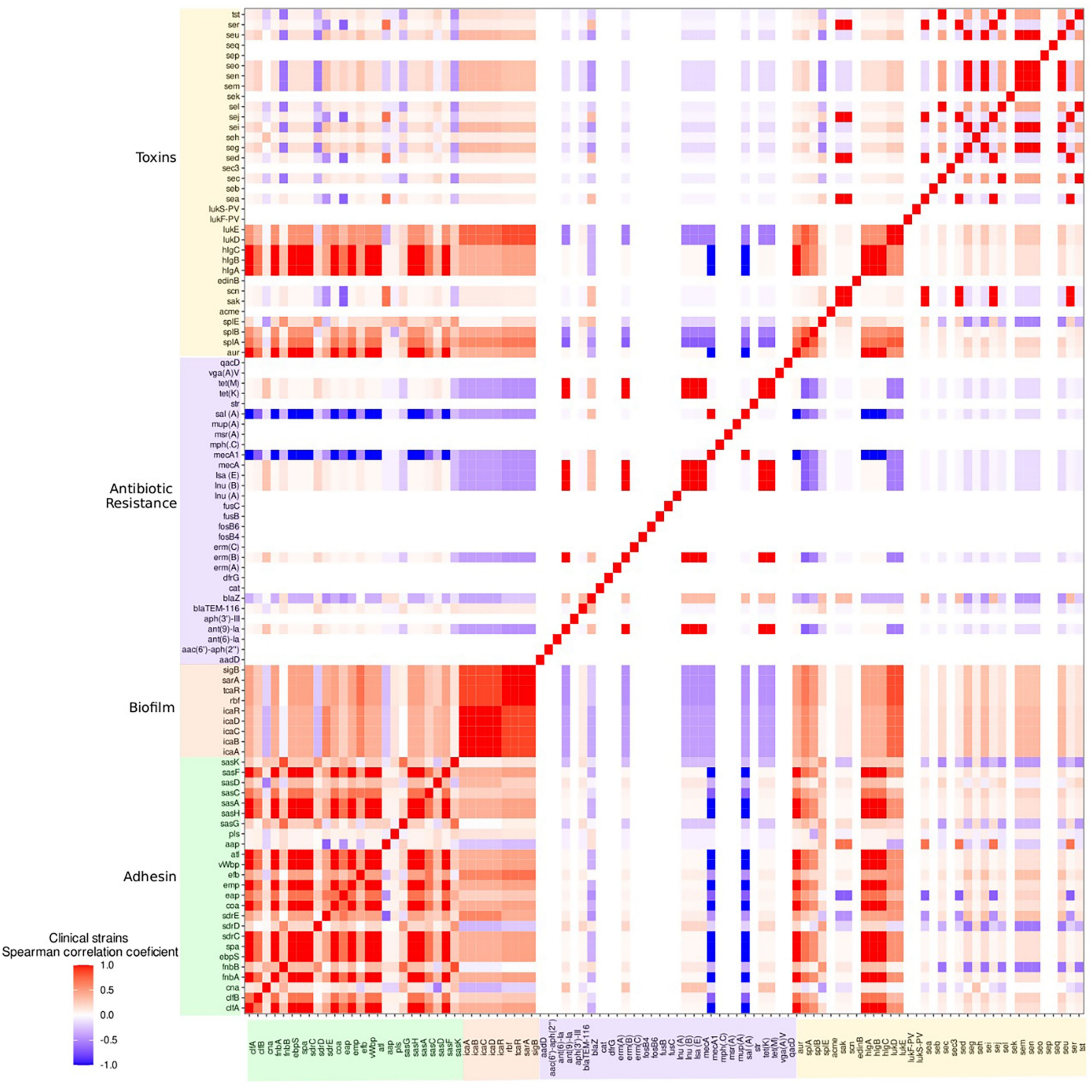
Heat map of the correlation, by the Spearman test, of the presence of *S. aureus* adhesion, biofilm, toxin, and antimicrobial resistance genes in clinical mastitis isolates.

With *S. aureus* isolates from clinical mastitis cases, there was a correlation, close to or equal to 1, between the *clfA, fnbA, ebpS, spa, sdrC, coa, emp, vWbP, atl, sasH, sasA, sasF adhesin genes,* and the *aur, hlgA, hlgB* and *hlgC* toxin genes (Figure 6). As well, there was a close association between the *fnbB*, *sasK* and *sasG* adhesin genes from this source. In the raw milk isolates, a high correlation was observed with the *sdrD*, *sasK, fnbA* and *splE* genes. In human isolates, there was a high correlation of the adhesion genes *efb*, *sdrC* and *sasG* and in skin infections and *sasG* and *sdrE* and in abscess isolates. All biofilm formation genes showed a high correlation with *S. aureus* from clinical and subclinical mastitis cases. In addition, the *icaADBC* operon and the *icaR* regulator were highly correlated in raw milk isolates and skin infections (Figure 6; Supplementary Material 4).

Antimicrobial resistance genes *ant(9)-Ia, erm(B), mecA, lsa(E), inu(B), tet(M)* and *tet(K)* as well as toxin genes *sak, scn, sea, sed, sej* and *ser* were highly correlated in the clinical mastitis group. As well, there was a correlation between the *seu, seg, sei, sen, sem* and *seo* genes in all groups. There was a correlation between the *sel, sec,* and *tst* gene in all bovine isolates. Further, the *sej, sed* and *ser* genes were associated with subclinical mastitis and abscess cases; the *seq* and *sek* genes with skin infections; *lukF-PV* and *lukS-PV* with subclinical mastitis, skin, and pus infections; *lukD* and *lukE* with abscesses and skin infections; *splA* and *splB* with abscesses; and the *lukD*, *lukE splA* and *splB* genes with pus isolates (Supplementary Material 4).

### Genome-wide association test accounting for population structure

TreeWAS analysis indicated that the *scn* gene was significant considering the Score 1 – “Terminal Score” which measures the measures sample-wide association across the leaves of the phylogenetic tree. The *ant(6)-la, lukS-PV, lukF-PV, sak and scn* were significant at the Score 2 – “Simultaneous Score” which measures the degree of parallel change in the phenotype and genotype across branches of the tree. None of the genes tested were significant at the Score 3 – “Subsequent Score” which measures the proportion of the tree in which the genotype and phenotype co-exist. These genes were further found to be significant with the multinomial logistic regression, alongside other ones mentioned above (Supplementary Material 5).

## Discussion

Adhesion is a crucial step in *S. aureus* colonization of the host and occurs before cell penetration, internalization, and chronic infection (Josse et al., 2017). The development of biofilms aids in evasion of host antimicrobial components and facilitates the exchange of genetic material (Idrees et al., 2021). Adhesin and biofilm genes *clfA, clfB, fnbA, ebpS, spa, coa, eap, emp, vWbp, atl, sasH, sasA, sasC, sasF, icaR, rbf, tcaR, sarA, sigB* and *icaADBC* were observed in >90% of the isolates. On the other hand, toxin and antimicrobial resistance genes were uncommon consistent with the notion that adhesion and biofilm formation play major roles in the pathogenesis of *S. aureus* (Petrie et al., 2020). In this regard, the *clfA* and the *clfB* genes, reported to promote microbial internalization in bovine mastitis, were present in almost all strains (Ying et al., 2021). Also, the *ebpS* gene, related to binding to elastin peptides in the host (Downer et al., 2002) was also very common (>95%). Similarly, the *fnbA* and *fnbB* genes responsible for adherence to immobilized elastin (Roche et al., 2004) were present in all groups, but *fnbB* gene with higher frequencies (>90%) in skin and pus infections. The high levels of expression of this gene are associated with the ability of *S. aureus* to internalize bovine mammary epithelial cells (Pereyra et al., 2016). Further, it was observed that *fnbB* was associated with *sdrD, sasG* and *sasK* in raw milk strains, and with *sasC* and *sasK* in clinical mastitis isolates (Supplementary Material 4). This is potentially an important association as the *sasG* and *sdrD* genes are reported to be involved in adhesion to epithelial cells (Roche et al., 2003; Abdelbary et al., 2020) and they play a role in cell aggregation and biofilm formation (Schroeder et al., 2009). In addition, our findings reinforce the recent observation that the *fnbB* gene is one of the main biofilm markers in *S. aureus* (Kadkhoda et al., 2020) and suggest that the *sasK* gene may also play an important role as well. The frequency of *sdrC*, *sdrD* and *sdrE* was lower in skin isolates in comparison with the other sources and *sdrD* was present less frequently in samples obtained from cattle. Thus, the *sdr* genes may not be important for epithelial tissue colonization and the *sdrD* gene may not play a key role in the adhesion to mammary gland and the establishment of bovine mastitis; however, further experiments are needed to test this hypothesis *in vivo.*

The adhesin genes *sasC, sasA, sasF* and *sasH* were present in >90% of the isolates from all sources. The *sasA* gene has been described in gangrenous mastitis and *sasF* in bovine mastitis isolates (Pizauro et al., 2021) but to date, little is known about *sasH* (Ythier et al., 2012). The observed lower frequencies (less than 50%) of the *cna*, *pls* and *aap* genes may be associated with the fact that the *cna* gene expresses a collagen-binding protein that is more often associated with skeletal muscle (Smeltzer and Gillaspy, 2000).The absence of *emp* and the presence of *pls* has been reported to be correlated with reduced virulence (Kurlenda et al., 2008), which was inversely observed (low frequency of *pls* and high of *emp*; Supplementary Material 2). The Emp and Eap proteins are also reported to be involved in *in vivo* biofilm formation and their expression is dependent on the *icaADBC* operon (Johnson et al., 2008). These genes, *emp* and *eap*, were observed in >90% of the strains in all sources and therefore, thought to be relevant in abscess formation and staphylococcal persistence. Also, in clinical mastitis isolates, the *vWbp* and *coa* genes showed a strongly correlation with each other (Figure 6), which is in agreement with the fact that these genes promote infection through prothrombin activation (Pickering et al., 2021).

The *aap* gene was present in less than 10% of isolates, whereas the *ica* locus and the *rbf*, *tcaR*, *saA* and *sigB* genes were present in >90% of the strains studied, except for clinical mastitis isolates (Supplementary Material 2). These differences may be related to the fact that PIA is the main exopolysaccharide synthesized by the *icaADBC* operon and that biofilm formation is less important in acute disease than in the maintenance of the strains in the host (Arciola et al., 2015). The association between *icaA*, *icaB*, *icaC*, *icaD,* and *icaR* was seen in strains isolated from raw milk and skin infections (Supplementary Material 4). Here, we identified that *sasG* and *fnbB* were very common in all groups evaluated (Figure 4) with the former having a frequency > 90% in abscess and pus isolates. Both *sasG* and *fnbB* were associated with invasive disease (Rasmussen et al., 2013) and the results showed a low correlation between these genes and clinical mastitis and raw milk isolates, with no association with *rbf*, *tcaR*, *sarA* and *sigB* genes and *icaR* (Supplementary Material 4) suggesting the importance of surface proteins in biofilm formation (Vergara-Irigaray et al., 2009; Geoghegan et al., 2010). Previous findings reported a high frequency of association of adhesion (*ebpS, atl, pls, sasH* and *sasF*) and biofilm-related genes (*icaABCD*) in *Staphylococcus* spp. from clinical mastitis (Pizauro et al., 2021). These results were also observed in the present study in clinical isolates in comparison with other sources, supporting the hypothesis that a more complex adherence and persistence mechanism may be required for *S. aureus* to be able to infect and persist in the bovine udder. That said, the staphylococci pangenome of clinical and subclinical mastitis isolates is still open so more sampling is required for more comprehensive analyses.

The toxin genes *aur, hglA, hglB* and *hglC* toxin genes were the only ones present in >90% in all groups evaluated. These genes were associated with the *cflA, fnbA, ebpS, spa, sdrC, coa, emp, vWF, atl, sasH, sasA* and *sasF* adhesin genes in clinical mastitis isolates (Figure 5). Aureolysin (expressed by the *aur* gene) modifies the adhesion factor CflB and activates other proteases that potentiate the virulence of *S. aureus* (McAleese et al., 2001). The proteins expressed by the *hglA*, *hglB* and *hglC* genes are bi-component leukotoxins that can form pores in the cell membrane and, consequently, lyse cells (Staali and Colin, 2021). Accordingly, the association of these genes products in clinical mastitis may be important targets for the development of vaccines and therapeutic agents (Ahmad-Mansour et al., 2021). Another association observed was the enterotoxins *sec*, *sei*, *sen*, *sem*, *seo* and *seu* in all evaluated groups, their frequencies were also similar; which have been observed by other studies (Indrawattana et al., 2013; Schwan, 2019; Ren et al., 2020). Also, previous studies have shown that *see* and *sec, sel* and *tst* are frequently present in MRSA strains (Hu et al., 2011), but this relationship was not observed in the current study. An association between the *sec*, *sel* and *tst* genes was seen only in bovine isolates, where the frequency of the *mecA* gene was low. The importance of these toxin genes in bovine mastitis has been highlighted previously (e.g., Fang et al., 2019). The *lukD* and *lukE* genes showed high association with themselves in isolates from abscesses, skin infections and pus; they were also considered significant for all groups in logistic regression but had higher frequencies in bovine isolates (Figure 3; Table 1). The genes *lukF-PV* and *lukS-PV* were correlated in all groups that were present, which is consistent with the literature since their products are secreted before joining to form the PVL toxin (Panton-Valentine leukocidin; Kaneko and Kamio, 2004).

More antimicrobial resistance genes were observed in human than in bovine isolates. Notably, the aminoglycoside resistance genes (*aadD, aac(6′)/aph(2″), aadE, ant(9)-Ia, aph(3′)-III*), chloramphenicol [*cat(pC233)*], tetracyclines [*tet(K), tet(M)*], macrolides and lincosamines [*erm(A), erm(B), mph(C)*), *msr(A)*], trimethoprim (*dfrG*) and β-Lactams (*blaZ, mecA*) were detected mostly in human isolates. Nevertheless, bovine strains presented *β*-lactam (*blaZ*), macrolide (*mphC*), macrolide, lincosamide, and streptogramin B (*msrA*), aminoglycoside (*aadD*), and tetracycline (*tetK*), all that have been described before (Pizauro et al., 2019). Previous studies detected high prevalence of *aadD* genes, *aac(6′)/aph(2″), tet(M), msr(A), aph(3′)-III, erm(A)* and *erm(B)* in *S. aureus* from patients admitted to ICUs (Abiri et al., 2017); as well as the presence of *tet(K), tet(M), mph(C), msr(A)* and other genes in nosocomial strains have been reported (Lozano et al., 2012). These relationships were also observed in the present study, and the *tet(M)* gene showed a high correlation with the *erm(A), dfrG* and *ant(9)-Ia* genes in pus, skin and abscess isolates with the addition of the *cat(pC233)* gene. In summary, the results of this study reinforce the growing presence of resistance genes in human strains, while pointing to a relatively low level of resistance genes in strains of bovine origin (Sweileh, 2021). There was an association of *ant(9)-Ia, mecA, tet(M) and tet(K), erm(B), lsa(E)* and *lnu(B)* genes, (which confer resistance to aminoglycosides, β-lactams, tetracyclines and macrolides and lincosamides) in the clinical mastitis group (Figure 5). Similarly, the *ACME, msr(A)* and *mph(C)* genes in pus isolates showed a low frequency.

The phylogenomic analysis was consistent with zoonotic transmission that the *S. aureus* is known for (Silva et al., 2022). This could be attributed to the evidence that suggests that some *S. aureus* strains are adapted to colonize and infect certain host species, whilst other lineages are non-specific (Schmidt et al., 2017). This behavior was highlighted in this study (Figure 5) with a high frequency observed for ST152 in skin lesions, ST 59 in pus suggesting their host specificity. Nevertheless, it was possible to observe that ST97 is predominantly associated with clinical mastitis, subclinical mastitis, and raw milk isolates and ST8 is predominantly associated with abscess and pus which indicates that they may be non-specific. In addition, some strains, from different origins share the same ST (e.g., ST8, ST15, and ST97; Figure 5). Bovine isolates were frequently ST97 and ST151, while most ST8, ST59, ST152, ST239 were from human sources. That said, these sequence types have been associated with both hosts consistent with zoonotic transmission in previous studies (Ndahetuye et al., 2021). The ST59, ST239, ST5, ST228, ST630, ST30, ST80, ST45 and ST88 were often from human cases in the current study. ST59 has been frequently observed in the community and more frequently in food samples (Pang et al., 2020). ST239 is globally disseminated and resistant to many antimicrobials commonly used in hospitals (Wang et al., 2014). As well, ST228, has high transmissibility (Abdelbary et al., 2020), while ST630 has high pathogenicity (Zong et al., 2020) and ST45 responsible for serious invasive diseases (Effelsberg et al., 2020). ST80 and ST88 seem to be mainly associated with the community (Stam-Bolink et al., 2007) and with animals, food and livestock ranchers (Otalu et al., 2018). ST151, ST126, ST479, ST20, ST71, ST133, ST504, ST115, ST425, ST737 and ST3183 were identified only in bovine isolates. ST151 has been frequently reported as bovine lineage common in cases of mastitis, susceptible to the acquisition of vancomycin resistance genes (Guinane et al., 2008); similar to the reported distribution of ST126 in bovine milk (Silva et al., 2016). Mainly described as clinical and subclinical mastitis strains, ST71 belongs to the same lineage as ST97, with more and more isolates demonstrated to be MRSA (Cormican and Keane, 2018), and in the present work they were present in bovine isolates. ST479 was only seen in milk samples (Chenouf et al., 2021); ST115 was consistent with bovine isolates (Smith et al., 2005) and ST3183 was recently identified in raw milk samples (McMillan et al., 2016). ST20 was cited in strains bovines and humans (Aung et al., 2019), and ST133 was cited in several hosts and environmental samples (Roberts et al., 2013). ST425 is currently characterized as a lineage associated with humans and animals (Paterson et al., 2014). Finally, ST737 was reported to be associated with the community, hospital environment (Karbuz et al., 2017) and cattle (Türkyilmaz and Erdem, 2013).

Our findings suggest that the *cflA, fnbA, ebpS, spa, sdrC, coa, emp, vWF, atl, sasH, sasA* and *sasF* adhesion genes, as well the *aur, hglA, hglB,* and *hglC* toxin genes are associated with clinical mastitis, and thus could be useful for screening tests and as candidates for more effectives vaccines. Further, the surface protein genes *sasG* and *fnbB* appear to be necessary for the interaction of *S. aureus* with cattle. It is also notewothy that the *blaZ* and *mecA* genes are frequently present in pus isolates. Finally, some STs have an epidemiological relationship with the type of disease, also showing zoonotic behavior.

## Data availability statement

The original contributions presented in the study are included in the article/Supplementary material, further inquiries can be directed to the corresponding author.

## Author contributions

All authors listed have made a substantial, direct, and intellectual contribution to the work, and approved it for publication.

## Funding

This work was carried out with the support of the Coordination for the Improvement of Higher Education Personnel – Brazil (CAPES) – Financing Code 001.

## Conflict of interest

The authors declare that the research was conducted in the absence of any commercial or financial relationships that could be construed as a potential conflict of interest.

## Publisher’s note

All claims expressed in this article are solely those of the authors and do not necessarily represent those of their affiliated organizations, or those of the publisher, the editors and the reviewers. Any product that may be evaluated in this article, or claim that may be made by its manufacturer, is not guaranteed or endorsed by the publisher.
