# Supplementary material for: Comparative genomics study of Staphylococcus aureus isolated from cattle and humans reveals virulence patterns exclusively associated with bovine clinical mastitis strains
Source: Front Microbiol. 2022 Nov 7;13:1033675. doi: 10.3389/fmicb.2022.1033675 (PMC9676464; doi:10.3389/fmicb.2022.1033675)
Supplement: Supplementary file 4 [file Data_Sheet_1.PDF]

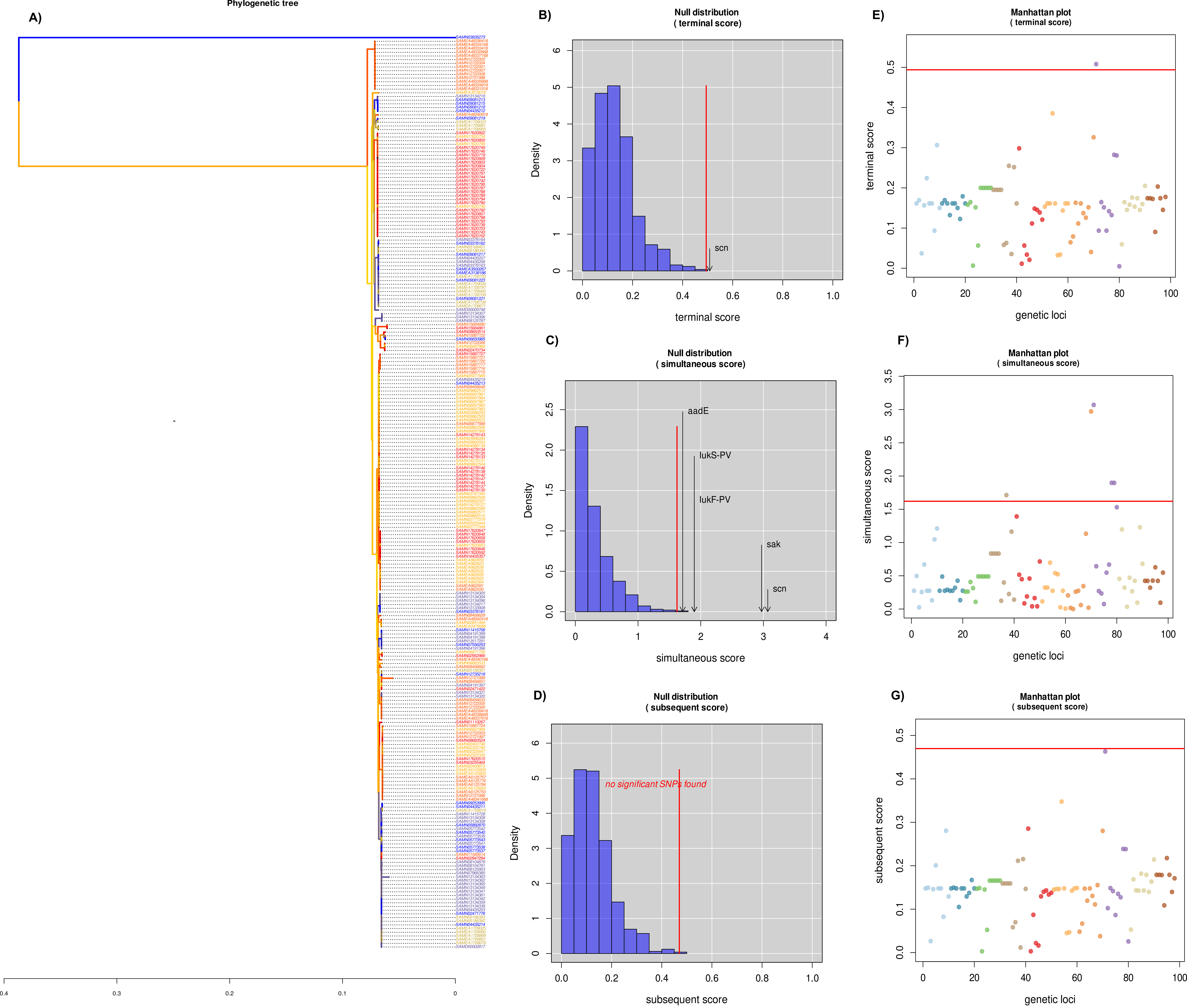

Supplementary material 5. treeWAS identified six genes that were significant thought the population structure, at the left, the phylogenetic tree constructed with the IQ-Tree and the phynotypes (Blue = Clinical Mastitis, Orange = Skin lesion, Yellow = Abcess, Red = Pus, Light blue = Subclinical mastitis and Brow = Raw milk. B-D Null distributions of simulated association scores for (B) Score 1, (C) Score 2, (D) Score 3, a significance threshold (red), above which real associated genes are indicated. E-G: Manhattan plots for (E) Score 1, (F) Score 2, (G) Score 3 showing association score values for all genes, a significance threshold (red), above which points indicate significant associations.
